# Clinical Assessment and Improved Diagnosis of Bocavirus-induced Wheezing in Children, Finland

**DOI:** 10.3201/eid1509.090204

**Published:** 2009-09

**Authors:** Maria Söderlund-Venermo, Anne Lahtinen, Tuomas Jartti, Lea Hedman, Kaisa Kemppainen, Pasi Lehtinen, Tobias Allander, Olli Ruuskanen, Klaus Hedman

**Affiliations:** University of Helsinki, Helsinki, Finland (M. Söderlund-Venermo, A. Lahtinen, L. Hedman, K. Kemppainen, K. Hedman); Helsinki University Central Hospital, Helsinki (L. Hedman, K. Hedman) Turku University Hospital, Turku, Finland (T. Jartti, P. Lehtinen, O. Ruuskanen); Karolinska Institute, Stockholm, Sweden (T. Allander); Karolinska University Hospital, Stockholm (T. Allander)

**Keywords:** Bocavirus, viruses, nasopharyngeal samples, serodiagnosis, respiratory infection, bronchiolitis, children, clinical assessment, Finland, research

## Abstract

Human bocavirus (HBoV) is a widespread respiratory virus. To improve diagnostic methods, we conducted immunoglobulin (Ig) G and IgM enzyme immunoassays with recombinant virus–like particles of HBoV as antigen. Acute-phase and follow-up serum samples from 258 wheezing children and single serum samples from 115 healthy adults in Finland were examined. Our assays had a sensitivity of 97% and a specificity of 99.5%. Of adults, 96% had immunity; none had an acute infection. Of 48 children with serologically diagnosed acute HBoV infections, 45 were viremic and 35 had virus in nasopharyngeal aspirates (NPAs). Of 39 HBoV NPA PCR–positive children co-infected with another virus, 64% had a serologically verified HBoV infection. HBoV caused illness of longer duration than rhinovirus and of equal severity to that of respiratory syncytial virus. Among children with bronchiolitis, >25% had acute HBoV infections. Accurate HBoV diagnosis requires serologic analysis or PCR of serum; PCR of NPAs alone is insufficient.

## Abstract 2

Accurate diagnosis of respiratory infections requires serologic analysis and PCR of serum.

A new parvovirus, human bocavirus (HBoV), was discovered during sequencing of respiratory tract samples from children. It has been detected worldwide in the nasopharyngeal tract, mainly in small children with lower respiratory tract infections (*1**,**2*). HBoV has been associated with upper and lower respiratory tract infections and shown to be a cause of pneumonia in children (*3**–**8*). Prolonged shedding of virus has been reported; >26% of children shed virus for 2 months, 4% for 3 months, and 2% for 4 months (*9*). Diagnosis of HBoV respiratory tract infections has been PCR based, leading to overrepresentation of HBoV co-infections with other respiratory viruses (*9**–**11*).

Along with others, we have shown that respiratory infections with HBoV elicit B-cell immune responses (*11**–**15*) and can be diagnosed serologically by using prokaryotic virus protein 2 (VP2) antigens in immunoblots (*11*). We report production in insect cells of VP2 of virus-like particles (VLPs) and their use in enzyme immunoassays (EIAs) for detection of HBoV-specific immunoglobulin (Ig) M and IgG in paired serum samples of pediatric patients with acute wheezing and in single serum samples of young healthy adults. Serologic results were compared with those of HBoV quantitative PCR (qPCR) of nasopharyngeal aspirates (NPAs) and paired serum samples of 258 children with complete sample sets. Clinical signs and symptoms of wheezing children with serologically verified acute HBoV infections with or without other respiratory virus infections (15 other viruses studied [*10*]) were compared with those of children infected with respiratory syncytial virus (RSV) or rhinovirus.

## Materials and Methods

### Patients and Samples

Acute-phase (at the time of admission) and convalescent-phase (2 weeks later) serum samples and NPA samples at the time of admission were obtained from 259 children (age range 3 months to 15 years, median 1.6 years) with acute expiratory wheezing (*10**,**16*). These children were tested by NPA PCR for 16 respiratory viruses (*10*); 117 of these children were tested by HBoV IgM and IgG immunoblots and HBoV serum qPCR (*10**,**11*). All remaining serum samples, except 1 convalescent-phase serum sample that was depleted, were tested by HBoV qPCR specific for the nucleoprotein 1 gene as described (*11*); all serum samples were tested by EIA. For 93 of these 258 children, follow-up serum samples were obtained 5–8 years later. In addition, 115 serum samples from healthy medical students were collected after informed consent was obtained. The study was reviewed and approved by the Ethics Committees of Turku and Helsinki University hospitals.

### Expression of VP2

**Figure 1:**
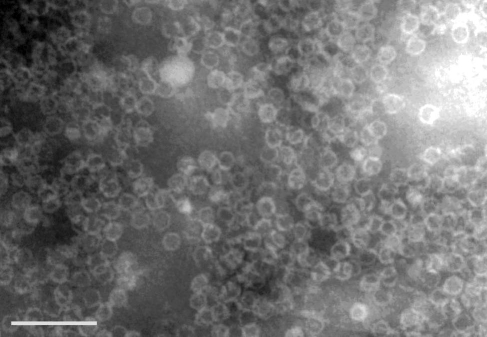
Recombinant human bocavirus virus protein 2 virus-like particles. Scale bar = 100 nm.

The putative major virus capsid protein VP2 gene (nt 3443–5071) of the HBoV St 2 isolate (GenBank accession no. DQ000496) was cloned into a baculovirus vector pAcSG2 (Becton Dickinson Biosciences, Franklin Lakes, NJ, USA) by standard procedures and confirmed by sequencing. The VP2-containing vector was transfected into Sf9 insect cells by using FuGENE 6 Transfection reagent (Roche, Basel, Switzerland). Two million adherent cells in T25 bottles were transfected in 1 mL of Insect Express media (Lonza, Basel, Switzerland) with a mixture of 2 μg plasmid, 250 ng linearized baculoGold DNA (Becton Dickinson Biosciences), and 15 μL FuGENE reagent. Fresh cells were infected 3 times every third day by using virus medium collected from the previous infection. VP2-containing Sf9 cells were harvested on day 3, and cell pellets were resuspended in phosphate-buffered saline (PBS), pH 7.5, at a concentration of 2.1 × 10^7^ cells/mL. Protease inhibitor (complete EDTA-free; Roche) was added (≈75 μL/mL), and cells were lysed by sonication (4 × 20 s). After subsequent centrifugation at 13,200 rpm for 3 min, VLPs were purified by 48-h CsCl gradient ultracentrifugation at 24,200 rpm (L-70 Ultracentrifuge; Beckman, Fullerton, CA, USA) at 4°C after fraction collection and dialysis against PBS. The product was concentrated in columns (Amicon Ultra-15 50,000 MWCO; Millipore, Billerica, MA, USA). Expressed HBoV VP2 was confirmed by sodium dodecyl sulfate–polyacrylamide gel electrophoresis to have a molecular mass of ≈60 kDa and by electron microscopy to be spherical symmetric parvovirus-like particles ≈20 nm in diameter (Figure 1). Before use as antigen, VLPs were biotinylated as described (*17*) .

### Serologic Analysis

The HBoV IgG EIA was conducted as described for parvovirus B19 (*18*) except that biotinylated VLPs were used at concentrations of 60 ng/well. Cutoff values were calculated from IgG EIA absorbances of the first serum sample obtained at admission of 34 children who had no IgM or maternal (waning) IgG against HBoV and showed a >4-fold increase of IgG against HBoV during follow-up testing. Cutoff absorbances for negative and positive IgG EIA results were 0.154 (mean + 3 SD) and 0.188 (mean + 4 SD), respectively.

For IgM EIA, a μ-capture format was used (*19*). Serum samples diluted 1:200 in PBS and 0.05% Tween (PBST) were applied in duplicate into wells of plates coated with goat anti-human IgM (Cappel/ICN Biomedicals, Costa Mesa, CA, USA) for 60 min at room temperature. After being rinsed 5 times with PBST, biotinylated HBoV VLPs were applied at a concentration of 25 ng/well and incubated for 45 min at 37°C. Bound antigen was visualized by using horseradish peroxidase–conjugated streptavidin (Dako, Glostrup, Denmark) at 1:12,000 in PBST plus 0.5% bovine serum albumin for 45 min at 37°C, followed by *o*-phenylenediamine dihydrochloride (Dako) and H_2_O_2_ for 15 min at 37°C. Cutoff values were calculated from IgM EIA absorbances of 5-year follow-up samples of 61 children who were IgG positive 5 years earlier. Cutoff absorbances for negative and positive IgM EIA results were 0.136 (mean + 3 SD) and 0.167 (mean + 4 SD), respectively. Parvovirus B19 serologic analysis was conducted by using commercial (Biotrin, Dublin, Ireland) and in-house EIAs (*18**,**20*).

### Statistical Analysis

Because most continuous data were skewed (by Kolmogorov-Smirnov test), they were analyzed by using regression analysis and generalized linear models after logarithmic transformation. Logistic regression analysis was used for categoric data. Statistical analyses were conducted by using SAS/STAT(r) software version 9.1.3 SP4 (SAS Institute Inc., Cary, NC, USA).

## Results

### qPCR

Complete sets of HBoV qPCR results for NPAs and serum samples were available for 258 children (Table 1). Among these children, 49 (19%) showed viremia in the first serum sample (29/49 [59%]), the second sample (12/49 [24%]), or both samples (8/49 [16%]). Most children who showed viremia in the first sample (62%) or both samples (87%) had a high (>10^4^ copies/mL) HBoV-DNA load in NPAs; most children who showed viremia only in the second sample (58%) had DNA-negative NPAs. These findings suggest a very recent infection. HBoV DNA loads in serum samples ranged from 112 copies/mL to 600,000 copies/mL and did not correlate with NPA DNA loads. Forty-nine (19%) of 258 children had NPAs that were PCR positive, but only 34 (13%) had both NPAs and serum samples that were PCR positive; 194 (75%) children had negative PCR results for both sample types (Table 1). Of long-term follow-up serum samples, only 1/93 was PCR positive; this sample was from a child who was seronegative 5 years earlier and seroconverted during follow-up. All 115 adult serum samples were HBoV PCR negative.

**Table 1:** Human bocavirus EIA and quantitative PCR results for 258 wheezing children, Finland*

PCR result	No. children	Serodiagnoses, IgM+,† no (%)						No serodiagnoses, no. (%)			
		SDG	SC	SI	IgG+	IgG–		All	IgG+	IgG–	Maternal
NPA+ serum+	34	33 (97)	21	4	6	2		1	0	1	0
NPA+ serum–	15	2 (13)	0	0	2	0		13	10	3	0
NPA– serum–	194	1† (0.5)	1†	0	0	0		193	100	88	5
NPA– serum+	15	12 (80)	6	1	2	3		3	1	2	0
NPA+	49	35 (71)	21	4	8	2		14	10	4	0
NPA+, high load	28	27 (96)	21	3	2	1		1	0	1	0
NPA+, low load	21	8 (38)	0	1	6	1		13	10	3	0
Serum+	49	45 (92)	27	5	8	5		4	1	3	0
NPA–	209	13 (6)	7†	1	2	3		196	101	90	5
Serum–	209	3 (1.4)	1†	0	2	0		206	110	91	5
Any PCR+	64	47 (73)	27	5	10	5		17	11	6	0
Total	258‡	48 (19)	28	5	10	5		210	111	94	5

*EIA, enzyme immunoassay; Ig, immunoglobulin; serodiagnoses (SDG), IgM positive and/or IgG conversion or increase; no serodiagnoses, IgM negative and no seroconversion/increase; SC, seroconversion; SI, serologic increase (>-fold increase in IgG); IgG+, constant level (<4-fold increase) of IgG in paired serum samples; IgG–, IgG negative in both serum samples; NPA, nasopharyngeal aspirate; Serum+, PCR positive in either or both serum samples. †One child was IgM negative. The IgG value of the convalescent-phase serum sample barely exceeded the cutoff value and the child was considered as not having an acute human bocavirus infection. ‡One of 259 children (*10*) was not included in this comparison because of depletion of the convalescent-phase serum sample before the PCR (NPA and acute-phase serum samples were PCR negative; both serum samples were IgG positive and IgM negative, indicating immunity).

### Antibody EIAs

**Figure 2:**
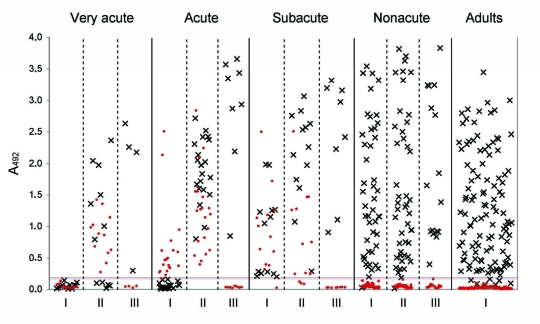
Scatter plots of individual absorbance values at 492 nm (A_492_) of immunoglobulin (Ig) G (×) and IgM (red dots) against human bocavirus (HBoV) in enzyme immunoassays (EIAs) for acute-phase (I), convalescence-phase (II), and 5-year follow-up (III) serum samples from wheezing children and single serum samples from young healthy adults, Finland. The 45 children with confirmed acute HBoV infections (by viremia and serodiagnosis) were divided into 3 groups according to the degree of acuteness (very acute, acute, and subacute) on the basis of findings in I and II serum samples. Very acute, I sample seronegative but II sample IgM positive (n = 12); acute, I sample IgM positive but IgG showed seroconversion (n = 20); subacute, IgG positive with a diagnostic increase or constant level in I and II samples, IgM positive (n = 13). Also shown are results for children without viremia or serodiagnosis (nonacute [for only the first 45 children with seropositive samples]), and young healthy adults (n = 115). EIA cutoffs are indicated by a blue line (IgG; 0.188) or a red line (IgM; 0.167). Dots below the cutoff lines indicate samples with absorbance values less than the negative cutoff values (i.e., IgM­– and IgG– results).

Of 258 wheezing children, 111 (43%) had serologic evidence of past infection; 48 (19%) of acute primary HBoV infection (Table 1; Figure 2). Of the latter group, 32 had detectable IgM with either IgG conversion (27/48) or a diagnostic increase (5/48); a total of 15 had IgM with no IgG (5/48) or with a constant IgG (10/48) absorbance value. A girl 1 year of age showed seroconversion in a convalescent-phase serum sample weakly positive for IgG but she had no IgM in either sample (Table 1). All other children who showed seroconversion or an increase in IgG were IgM positive. Incidence rates of serologically verified, acute, primary HBoV infection among wheezing children varied with age, with a peak of 28% in children 1–<2 years of age (Table 2). Prevalence of HBoV immunity increased with age, reaching 100% at 7 years of age (Table 2). This study included 27 (10%) of 258 children <6 months of age (median 4.8 months, mean 4.7 months). Eight (30%) of 27 children were IgG positive; IgG in 7 of these children was presumably of maternal origin. One infant (3.7%), a 3.6-month-old boy, had a serologically verified acute HBoV infection.

**Table 2:** Incidence rates of serologically verified acute human bocavirus infections and prevalence rates of bocavirus immunity among 258 wheezing children, Finland*

Characteristic	Age group							
	<6 mo	6–<12 mo	1–<2 y	2–<3 y	3–<4 y	4–<7 y	7–<15 y	Total
Incidence	1/26 (4)	9/37 (24)	25/88 (28)	5/42 (12)	3/25 (12)	2/19 (10)	0/14 (0)	45/251 (18)
Prevalence	1/21† (5)	5/37 (13)	28/88 (32)	30/42 (71)	16/25 (64)	16/19 (84)	14/14 (100)	110/246† (45)

*Values are no. positive/no. tested (%). Discrepant results of enzyme immunoassay and PCR results from 7 children were not included (see Table 1 and text). †Five infants had maternal antibodies (defined as waning immunoglobulin G absorbance values in paired serum samples). Two other infants became seronegative and were considered virus negative.

Comparison of qPCR and EIA results for 258 children is shown in Table 1. Among 49 children who showed viremia, 45 (92%) had a serodiagnosis of HBoV infection: i.e., had IgM or an increase in IgG. Of the remaining 4 viremic children, 1 was seropositive and 3 were seronegative; 2 of the seronegative children showed viremia in a second sample, possibly indicating a very acute infection (their NPA samples at the time of admission had been PCR negative). Among 209 nonviremic children, 206 (99%) showed nondiagnostic serologic results (Table 1). Only 3 (1.4%) of 209 children had a serodiagnosis: the 1-year-old girl with an apparent false seroconversion described above and 2 children with PCR-positive NPAs and a positive result for IgM but a constant IgG level, which suggests a subacute infection.

Of 49 (19%) of 258 HBoV NPA PCR-positive children, 35 (71%) had a serodiagnosis, 33 of whom were also viremic (Table 1). Of 28 patients with a high load of HBoV DNA in NPAs, 27 (96%) had an HBoV serodiagnosis, compared with only 8 (38%) of 21 with a low DNA load. Conversely, among 209 children without HBoV DNA in NPAs, 13 (6%) had a serodiagnosis, of whom 12 were also viremic.

Among 34 (13%) of 258 children who were HBoV PCR positive by both NPAs and serum samples, 33 (97%) had a serodiagnosis; the remaining child (a girl 6 months of age) was seronegative and viremic only in the second sample, which indicated a very acute infection. In contrast, of 194 (75%) of 258 children who were HBoV PCR negative in both NPAs and serum, only 1 (0.5%) had a serodiagnosis (the IgM-negative 1-year-old girl). Conversely, of 48 (19%) of 258 children who had a serologically diagnosed acute HBoV infection, 35 (73%) were PCR positive for NPAs, 45 (94%) were viremic, and 47 (98%) were PCR positive for NPAs or serum (Table 1). If one considers a positive PCR result for serum as the standard for diagnosis (n = 258), our EIA had a sensitivity of 92%, a specificity of 99%, and a positive predictive value of 94%. If PCR positivity for NPAs and serum is the standard for diagnosis (n = 128), the sensitivity is 97%, the specificity is 99.5%, and the positive predictive value is 97%.

All 258 children had been tested for 16 respiratory viruses (*10*). Of 12 (4.6%) with a serologically diagnosed acute HBoV single infection, 12 were viremic and 10 (83%) were HBoV DNA positive in NPAs (all with high DNA loads). Among 39 HBoV NPA PCR-positive children co-infected with 1 or 2 other respiratory viruses (rhinovirus, enterovirus, RSV, adenovirus, influenza A virus, or parainfluenza virus), 25 (64%) had a serologically diagnosed primary HBoV infection; 17 (94%) of 18 with a high HBoV DNA load and 8 (38%) of 21 with a low HBoV DNA load had primary infections. Among viremic children with serologically confirmed acute HBoV infections, 33 (73%) of 45 had co-infections compared with 12 (92%) of 13 children with HBoV DNA in NPAs but without serodiagnosis or viremia; this difference was not significant (p = 0.26).

Follow-up serum samples obtained 5–8 years later were available for 93 of 258 children. Of 41 IgG-negative children, 38 (93%) had seroconverted, and all 21 acutely infected and all initially IgG-positive children were still IgG positive and had no IgM or HBoV DNA. Of 115 young adults, none had IgM, 110 (96%) had IgG (including 2 with borderline results), and none were viremic. Absorbance values are shown in Figure 2.

Serodiagnostic findings for infection with parvovirus B19 (IgM positive or low epitope-type specificity index) (*18**,**20*) were not observed among children with a serodiagnosis of HBoV infection. Among other children, 3 were IgM positive for parvovirus B19, of whom 1 was seronegative for HBoV and 2 were seropositive for HBoV.

### Clinical Characteristics

The 258 children tested for 16 viruses were analyzed for clinical characteristics. Median age of 46 children with acute HBoV infection diagnosed by serologic analysis and PCR of serum was 1.3 years (range 0.3–6.1 years), median age of 91 of 258 nonexposed seronegative children was 1.1 years (range 0.2–4.2 years), and median age of 110 of 258 children with HBoV immunity was 2.8 years (range 0.5–5.2 years) (p<0.0001).

Clinical data were compared among children infected only with HBoV (n = 12), rhinovirus (n = 56), RSV (n = 36), and HBoV and any other virus (n = 34) (Table 3), HBoV and rhinovirus (n = 14), and HBoV and RSV (n = 7). Among single infections, RSV induced wheezing earliest in life (median 0.8 years), followed by HBoV (1.4 years) and rhinovirus (2.1 years; p<0.0001). Age-adjusted comparison of single infections showed longer duration of hospitalization (p = 0.0069), longer duration of cough (p = 0.0012), and longer duration of cough before admission (p<0.0001) for patients with acute HBoV infections than for patients with rhinovirus infections. However, children infected with rhinovirus had a higher leukocyte count at the time of admission (p = 0.0002). When compared with patients infected with RSV, patients infected with HBoV showed longer duration of cough before admission (p = 0.019). Differences in clinical variables were not observed for rhinovirus or RSV, whether in children with single infections or those co-infected with HBoV. We found no differences between children co-infected with HBoV and 1 or 2 other viruses. Nonrespiratory symptoms, including diarrhea, were rare (Table 3).

**Table 3:** Clinical characteristics of pediatric patients with acute wheezing caused by HBoV, rhinovirus, RSV, or mixed virus infections*†

Factor						Between groups infected with			
	Virus infection					Single virus (n = 104)			Single + mixed (n = 46)‡
	HBoV (n = 12)	Mixed (n = 34)	Rhinovirus (n = 56)	RSV (n = 36)		p value§	Adjusted p value¶		p value§
Age, y	1.4 (0.8–3.2)	1.3 (0.3–6.1)	2.2 (0.4–12.5)	0.9 (0.3–4.5)		<0.0001	–		0.57
Male, no. (%)	9 (75)	23 (68)	36 (64)	20 (56)		0.45	0.073		0.64
At admission									
Severity of illness, scale 0–12	7 (4–10)	7 (2–10)	6 (2–10)	7 (4–10)		0.057	0.15		0.43
% Oxygen saturation	97 (88–99)	96 (91–99)	96 (88–100)	96 (89–99)		0.98	0.97		0.95
Temperature, °C	37.6 (36.1–39.1)	37.7 (36.2–39.5)	37.4 (36.2–39.3)	37.9 (36.3–40.1)		0.0014	0.032		0.71
CRP, mg/L	7.50 (0–78)	10 (0–45)	18 (0–191)	8 (0–96)		0.25	0.81		0.48
Leukocyte count, × 10^9^/L	8.50 (6.3–11.9)	11.1 (5.1–23.6)	12.1 (5.6–20.8)	9.4 (4.9–20.7)		0.0003	<0.0001		0.029
Duration of hospitalization, h	30 (18–78)	27 (6–90)	18 (6–74)	38 (6–138)		<0.0001	0.0066		0.12
Duration of cough, d	15 (4–66)	11 (2–38)	8 (1–36)	11 (4–22)		0.0022	0.020		0.062
Before admission	5 (1–60)	3 (1–28)	2 (0–19)	4 (1–14)		<0.0001	<0.0001		0.038
After hospitalization	6 (2–14)	5 (0–14)	6 (0–14)	4 (0–13)		0.40	0.42		0.36
Moderate–severe after hospitalization	1 (0–8)	1 (0–14)	2 (0–14)	0 (0–4)		0.019	0.0052		0.34
Duration of breathing difficulty, h	4 (1–9)	4 (1–11)	3 (0–36)	6 (1–14)		<0.0001	0.0047		0.48
Before admission	1 (0–7)	1 (0–7)	1 (0–19)	2 (0–6)		0.040	0.67		0.63
After hospitalization	0 (0–4)	1 (0–10)	0 (0–14)	3 (0–11)		0.028	0.071		0.40
Moderate–severe after hospitalization	0 (0–0)	0 (0–6)	0 (0–14)	0 (0–1)		0.39	0.49		0.13
Other symptoms, no. (%) patients									
Acute otitis media	4 (33)	16 (47)	16 (29)	26 (72)		0.0003	0.073		0.41
Nonrespiratory symptoms									
Diarrhea	1 (8)	4 (12)	2 (4)	7 (19)		0.072	0.21		0.74
Balance problems	0	1 (3)	2 (4)	2 (6)		0.92	0.53		0.45
Rash	0	0	2 (4)	0		1.00	1.00		–
Arthritis or arthralgia	0	0	0	0		–	–		–

*Mixed infections consisted of HBoV plus >1 virus. Values are median (range) except as indicated. HBoV, human bocavirus; RSV, respiratory syncytial virus; CRP, C-reactive protein. †Analysis by regression analysis using generalized linear models after logarithmic transformation for continuous data or by logistic regression analysis for categorical data. Significant intergroup differences were found when persons with HBoV infections were compared with those with other infections: age: HBoV vs. RSV, p = 0.036; leukocyte count: HBoV vs. rhinovirus, p = 0.0009 unadjusted and p = 0.0002 adjusted; duration of hospitalization: HBoV vs. rhinovirus, p = 0.0027 unadjusted and p = 0.0069 adjusted; duration of cough: HBoV vs. rhinovirus, p = 0.0009 unadjusted and p = 0.0012 adjusted; duration of cough before admission: HBoV vs. rhinovirus, p<0.0001 unadjusted and p<0.0001 adjusted and HBoV vs. RSV, p = 0.028 unadjusted and p = 0.019 adjusted; acute otitis media: HBoV vs. RSV, p = 0.0005 unadjusted. ‡A child with 1 PCR-positive nasopharygeal aspirate and a PCR-negative serum sample was classified as having a subacute HBoV infection. In Table 1, he was 1 of 3 nonviremic children with a serodiagnosis for HBoV infection. §Unadjusted. ¶Adjusted for age.

Acute HBoV infection was found in children with bronchiolitis. Among children <2 years of age, acute HBoV infection was detected in 26 (27%) of 95 children having their first wheezing episode and in 35 (25%) of 141 children having their first or recurrent wheezing episode, excluding asthmatic children (defined as children considered for initiation of daily long-term control therapy according to the recent US guidelines for diagnosis and management of asthma [*21*]). Children with HBoV and RSV single infections showed a similar overall severity of illness (median 7, range 4–10 on a scale of 0–12), whereas acute otitis media (AOM) was more frequent among children with RSV single infections (p = 0.0005) (Table 3).

## Discussion

HBoV infections have been commonly diagnosed by PCR of respiratory tract samples. Only a few serologic studies have been reported; these studies have addressed mainly epidemiologic issues (*11**–**15*). However, Endo et al. documented seroconversions by immunofluorescent analysis in 4 HBoV PCR-positive patients (*12*), and Lindner et al. detected IgM against HBoV by EIA in 12 patients, 10 of whom were viremic children (*15*). In a study of 117 wheezing children, we showed by using immunoblotting and prokaryotically expressed HBoV VP2 capsid antigens that HBoV infections can be diagnosed serologically (*11*). We also showed that the unique region in VP1 is are less immunogenic than the major virus capsid protein VP2. We have now expressed HBoV VP2 VLPs in insect cells for use in IgM and IgG EIAs that are superior to immunoblots in diagnostic performance. We diagnosed acute primary HBoV infections in 48 (19%) of 258 children with expiratory wheezing. Consistent with other reports, no cross-reactivity between 2 human pathogenic parvoviruses (B19 and HBoV), was detected (*11**–**13**,**15*). Prevalence rates of immunity increased with age from 5% in infants to >64% in children 2–4 years of age and continued to increase until a maximum of 100% was reached in children 7 years of age. Seroprevalence among young adults was 96%. Furthermore, IgG levels of adults were as diverse as those of children (Figure 2). These results contrast sharply with our previous immunoblot results with denatured VP2 antigen (*11*), which showed decreased seropositivity among children >2 years of age. This difference is likely caused by a time-related conformational dependence of the antibody, similar to immunity to B19 virus (*18**,**20**,**22**,**23*). In other HBoV seroprevalence studies, similar rates were reported (*12**,**13**,**15*), which validate the accuracy of our results.

HBoV infection has been shown (by PCR of NPA samples) to be most prevalent in children 6 months to 3 years of age; adults are less affected (*8**,**24**–**30*). Consistent with this finding, the incidence of serologically verified acute HBoV infections in our study was highest (28%) during the second year of life; median age of children with acute HBoV infections was 1.3 years. Only 2 children were infected at >4 years of age; 1 of them was seronegative. Children <6 months of age might be protected from infection by maternal antibodies. Rapidly decreasing seroprevalence rates from ≈90% in infants <3 months of age to ≈5% in infants 6 months of age were reported (*12**,**13*). In our study, 26% of 27 children <6 months of age (including 1 infant <3 months of age) had maternal antibodies, and 1 child in this age group had an acute HBoV infection.

When we compared serologic results with those of PCR, we found a profound difference between NPA PCR results and serum PCR results. Although results of serodiagnosis were identical to results of serum analysis by PCR, only 71% of NPA-positive children and 6% of NPA-negative children had an HBoV serodiagnosis. However, all but 1 (96%) of the children with a high load of HBoV DNA in NPAs had a serodiagnosis, compared with only 38% of those with a low DNA load. This finding supports the view that a low HBoV DNA load is not evidence of acute primary infection (*10**,**11**,**31**,**32*). Studies of consecutive NPA samples have shown that HBoV DNA can persist in the nasopharynx for several months (*9**,**33*). We also noted that 22% of children with HBoV DNA in their first serum sample continued to show viremia (with 2 logs less DNA) in the second serum sample obtained an average of 19 days later. That HBoV does not often persist in serum indicates that regardless of its magnitude, viremia is an excellent marker of acute HBoV infection.

The clinical role of codetection of HBoV and other viruses in NPAs has been questioned. It is not easy to determine whether such co-infections are sequential infections or simultaneous viral infections. Serologic analysis is a more precise approach for diagnosis of HBoV infections. When compared with PCR-positive results in serum and NPAs, diagnostic sensitivity and specificity of our antibody EIAs were as high as 97% and 99.5%, respectively; positive predictive value was 97%. We showed by using EIAs that among wheezing children, >60% of co-infections in children with HBoV NPA PCR-positive results, particularly in children with a high HBoV DNA load, are acute primary HBoV infections and should be considered in the diagnosis of respiratory disease. No differences in occurrence of co-infections were observed in children with a serologically confirmed diagnosis of infection with HBoV compared with children positive for HBoV by only PCR of NPAs.

Several groups have compared clinical features of HBoV infections with those of other respiratory virus infections. In those studies, diagnoses of infection with HBoV were based only on PCR positivity of NPAs, which as we showed, is not an ideal marker for detection of acute HBoV infection. We assessed clinical findings of our patients with serologically verified acute HBoV infections. Comparison of HBoV-induced wheezing with that induced by rhinovirus is notable because rhinovirus is commonly associated with wheezing in older children and has been recognized as a risk factor for recurrent wheezing and asthma in young children (*34**–**36*). Also notable is a comparison of HBoV and RSV because RSV is the dominant cause of bronchiolitis in infants (*37**,**38*). Our data showed that wheezing induced by RSV occurred at the youngest age (median 10 months), followed by that induced by HBoV (17 months), and rhinovirus (25 months). Age-adjusted comparisons showed that HBoV-infected children were hospitalized longer than rhinovirus-infected children. Illnesses after HBoV infection lasted longer than illnesses after rhinovirus infection. However, we did not demonstrate that co-infection with HBoV would increase illness duration or severity, as has been reported for rhinovirus- and RSV-induced bronchiolitis (*39*).

Children with HBoV co-infections seemed to have more AOM (47%) than those with single HBoV infections (33%), but this difference could be explained by inclusion of 7 RSV-positive children in this group. The highest rate of AOM (72%) was in children with RSV-induced wheezing. Alper et al. reported differences in frequencies of various respiratory viruses associated with AOM (HBoV was not included), but statistical significance was not achieved (*40*). We found a difference in the frequency of AOM between children with RSV-induced wheezing and those with HBoV-induced wheezing.

Serologically confirmed primary HBoV infections detected in 12 symptomatic children with no signs of other respiratory virus infections (by PCR, culture, antigen detection, or serologic analysis) demonstrate that HBoV is a cause of acute wheezing in young children. Moreover, the fact that acute HBoV infection was detected in 27% of hospitalized children who were <2 years of age when they had their first episode of wheezing indicates that HBoV is a causative agent of bronchiolitis with clinical severity comparable with that of RSV. HBoV respiratory infections can be diagnosed with moderate accuracy by qPCR of NPAs. However, the most reliable methods for diagnosis of acute symptomatic HBoV infection are PCR of serum samples and serologic analysis for IgM and IgG.
